# Halting the Canadian STRIDER randomised controlled trial of sildenafil for severe, early-onset fetal growth restriction: ethical, methodological, and pragmatic considerations

**DOI:** 10.1186/s13104-022-06107-y

**Published:** 2022-07-07

**Authors:** Peter von Dadelszen, François Audibert, Emmanuel Bujold, Jeffrey N. Bone, Ash Sandhu, Jing Li, Chirag Kariya, Youkee Chung, Tang Lee, Kelvin Au, M. Amanda Skoll, Marianne Vidler, Laura A. Magee, Bruno Piedboeuf, Philip N. Baker, Sayrin Lalji, Kenneth I. Lim

**Affiliations:** 1 Department of Women and Children’s Health, School of Life Course Sciences, King’s College London, London, UK; 2 Department of Obstetrics and Gynaecology, BC Children’s Hospital Research Institute, University of British Columbia, Vancouver, BC Canada; 3 Département d’Obstétrique-Gynécologie, Faculté de Médecine, Université de Montréal, Montréal, QC Canada; 4 Département d’Obstétrique, de Gynécologie et de Reproduction, Faculté de Médecine, Université Laval, Québec, QC Canada; 5 Département de Pédiatrie, Faculté de Médecine, Université Laval, Québec, QC Canada; 6 Pro-Vice-Chancellor Research and Enterprise, University of Leicester, Leicester, UK

**Keywords:** Fetal growth restriction, Sildenafil, Randomised controlled trial, Early trial halting

## Abstract

**Objectives:**

To determine the efficacy and safety of sildenafil citrate to improve outcomes in pregnancies complicated by early-onset, dismal prognosis, fetal growth restriction (FGR). Eligibility: women ≥ 18 years, singleton, 18 + 0–27 + 6 weeks’ gestation, estimated fetal weight < 700 g, low PLFG, and ≥ 1 of (i) abdominal circumference < 10th percentile for gestational age (GA); or (ii) reduced growth velocity and either abnormal uterine artery Doppler or prior early-onset FGR with adverse outcome. Ineligibility criteria included: planned termination or reversed umbilical artery end-diastolic flow. Eligibility confirmed by placental growth factor (PlGF) < 5 th percentile for GA measured post randomization. Women randomly received (1:1) either sildenafil 25 mg three times daily or matched placebo until either delivery or 31 + 6 weeks. Primary outcome: delivery GA. The trial stopped early when Dutch STRIDER signalled potential harm; despite distinct eligibility criteria and IRB and DSMB support to continue, because of futility. NCT02442492 [registered 13/05/2015].

**Results:**

Between May 2017 and June 2018, 21 (90 planned) women were randomised [10 sildenafil; 11 placebo (1 withdrawal)]. Baseline characteristics, PlGF levels, maternal and perinatal outcomes, and adverse events did not differ. Delivery GA: 26 + 6 weeks (sildenafil) vs 29 + 2 weeks (placebo); p = 0.200. Data will contribute to an individual participant data meta-analysis.

## Introduction

Women with pregnancies complicated by severe, early-onset fetal growth restriction (FGR) have limited options. A 50% chance of intact perinatal survival (i.e., survival without major complications of prematurity or perinatal asphyxia) requires both birth at least at 28 + 0 weeks’ and birth weight of at least 700 g [1]. Therefore, in the absence of effective pharmacological interventions, intensive fetal surveillance is employed to prolong pregnancies for as long as possible until either fetal or maternal deterioration requires delivery [2].

Antenatally, the performance of combined ultrasound estimates of fetal biometry and Doppler waveform indices to discriminate early-onset FGR due to placental dysfunction from constitutionally-small fetuses [2] can be improved by observing maternal plasma placental growth factor (PlGF) < 5th percentile for gestational age (GA) [3].

Sildenafil citrate vasodilates the myometrial arteries isolated from women with FGR-complicated pregnancies [4]. At BCWH, 10 women with pregnancies complicated by early-onset FGR (abdominal circumference [AC] < 5th percentile) and either the GA was < 25 + 0 weeks or an estimate of fetal weight was < 600 g (excluding known major fetal complications) took sildenafil (25 mg three times daily until delivery) [5]. Sildenafil treatment was associated with increased fetal AC growth, compared with 17 institutional sildenafil-naive early-onset FGR controls]) [5]. A trial of sildenafil in women with late-onset pre-eclampsia had shown evidence of neither benefit nor harm [6].

Therefore, following protocol design, local and national approvals, the formation of an independent Data and Safety Monitoring Board, and as part of an international consortium of STRIDER (Sildenafil TheRapy in dismal prognosis early onset fetal growth restriction) trials (in support of the Global Obstetric Network (GONet) [7]), a Canadian STRIDER randomised controlled trial was undertaken. This trial was unique from others in that a low PLGF was included in the eligibility criteria.

## Main text

**Fig. 1:**
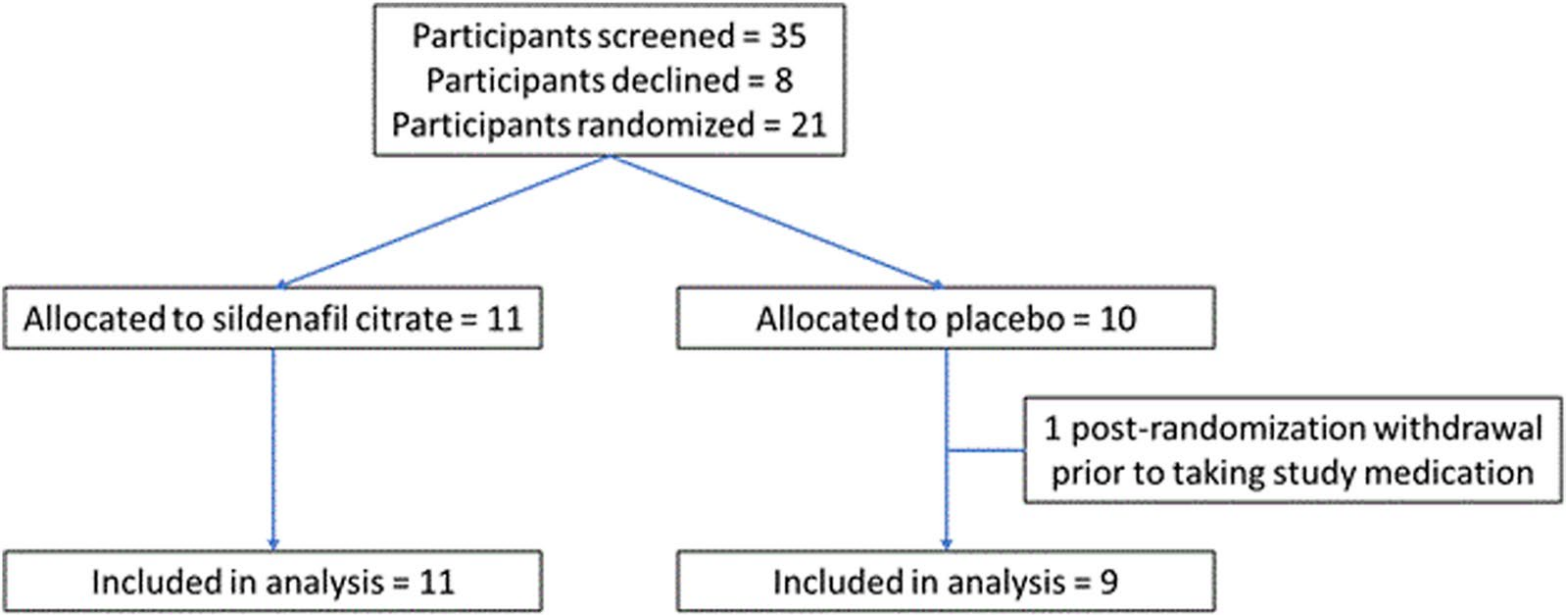
STRIDER Canada Trial Profile

The STRIDER Canada trial was a national multisite individual participant double-blind, placebo-controlled randomised controlled trial (NCT02442492 [registered 13 May 2015]). (Fig. 1. CONSORT diagram). The DSMB monitored the progress of the trial and all serious adverse events (SAEs). As the trial was halted early, only three sites became active, BC Women’s Hospital and Health Centre (BCWH’s, Vancouver), Ste Justine (Montréal), and Centre hospitalier universitaire de Québec (Québec).

### Recruitment

Women were eligible to be approached to provide written informed consent if they were aged ≥ 18 years with a singleton pregnancy at 18 + 0—27 + 6 weeks’ gestation, estimated fetal weight < 700 g, and at least one of (i) fetal AC < 10th percentile for GA (local criteria); or (ii) reduced fetal growth velocity (AC growth < 50% of anticipated using institutional fetal biometry charts) and either abnormal uterine artery Doppler or prior early-onset FGR with adverse perinatal outcome (defined as either a perinatal or infant death related to FGR or a life-altering complication of either prematurity or FGR).

Exclusion criteria were (i) decision made to terminate the index pregnancy; (ii) reversed end-diastolic flow by umbilical artery Doppler; (iii) prior participation in a STRIDER trial; (iv) maternal factors (e.g., pre-eclampsia or gestational hypertension [index pregnancy]; known HIV positive status [drug-drug interaction between sildenafil and antiretrovirals]; known significant maternal heart disease; current cocaine or crystal meth drug use; receiving prazosin, other peripheral alpha-blockers, or nitrates; or allergy to sildenafil); or (v) fetal factors (e.g., known aneuploidy, anomaly, syndrome, or congenital infection confirmed at enrolment).

Once consent had been obtained, post randomization eligibility was confirmed in real-time by Alere (San Diego, CA) Triage™ plasma PlGF < 5th percentile for GA [8]; for gestational ages 18 + 0–20 + 0 weeks, the 5th percentile was determined from samples from women attending the BCWH s EMMA (Evaluating Maternal Markers of Adverse placental outcomes) Clinic at 16 + 0–24 + 0 weeks who delivered normally-grown infants (10th–90th percentile for GA) at term without either hypertension or diabetes. The EMMA Clinic 5th percentile PlGF values were contiguous with the NORMALS cohort 5% percentile line [8].

### Randomisation

Randomisation was centrally controlled using the web-based platform integrated System for Trial Allocation and Randomisation (iSTAR; PRE-EMPT [PREgnancy Evidence, Monitoring, Partnerships & Treatment] team, BC Children’s Hospital Research Institute (BCCHR). Randomisation was stratified by centre with random blocks of 2 or 4. Women were randomised 1:1 to receive either sildenafil (25 mg three times daily) or matched placebo (three times daily). Each study drug bottle contained 30 over-encapsulated capsules (to mask arms), equivalent to a 10-day supply of either sildenafil or placebo. Sildenafil tablets were sourced from Pharmascience Inc (DIN 02,317,559). The Bay Area Health Trust (BARL), ON, made placebo capsules by over-encapsulation.

### Follow-up

STRIDER Canada was a pragmatic trial; in the absence of standardised care available at all participating centres, the Canadian STRIDER trial protocol allowed centres to provide their usual pattern of care. All women received enhanced fetal and maternal surveillance based on concurrent Society of Obstetricians and Gynaecologists of Canada guidance regarding the diagnosis management of FGR [9].

At enrolment, baseline data were collected regarding current and past medical history and demographics including ethnicity, past obstetric history, medication use, allergies, aneuploidy screening, invasive testing, and congenital infection results.

Fetal biometry (biparietal diameter, head circumference, AC, and femur length) and Doppler indices (ductus venosus; uterine, umbilical and middle cerebral arteries,) were collected from the latest pre-randomisation ultrasound scan. Similarly, data for maternal assessment prior to randomisation were collected for PlGF, complete blood count, renal (creatinine, urea) and liver function (aspartate and alanine transaminase, albumin), urinalysis, and blood pressure measurements. Doppler indices, PlGF testing, and blood pressure measurements were repeated at 48 h post-1st dose. All participants undertook weekly maternal and fetal assessments and PlGF testing. Other clinically-indicated care was undertaken as per local practice or at the discretion of a treating clinician. No special restrictions were required with regards to diet, activities, or other lifestyle items.

In addition, each participant was given a patient medication diary and were encouraged to note any missed doses, adverse events, and symptoms. The dairy and pill counts were reviewed by the study team during antenatal visits.

Decisions to deliver were made by the treating physicians according to local practice. If undelivered, all participants discontinued the study drug once they reached 31 + 6 weeks GA.

### Outcomes

Primary outcome: gestational age at delivery (days) (sildenafil vs placebo). The unit of analysis was ‘fetus’. Secondary outcomes: livebirth, survival to hospital discharge, intact discharge, and combined non-CNS severe morbidity.

### Stopping rule

Stopping rule: STRIDER Canada would be halted if two other STRIDER trials determined any potential benefit or harm before the STRIDER Canada trial was completed.

### Data management

All data were entered into a REDCap (Research Electronic Data Capture, Vanderbilt University) platform developed and co-ordinated on behalf of all the STRIDER trials by the PRE-EMPT team. This approach was to facilitate the a priori planned STRIDER consortium individual participant data (IPD) meta-analysis [10].

### Statistical analyses

Continuous variables were reported as means [95% confidence interval (CI)] for the between-arm mean difference. Categorical variables were presented as counts (percentages) and compared using chi-squared tests. P < 0.05 was considered statistically significant. Data were analysed in R statistical software (version 3.6.3; R Foundation for Statistical Computing, Vienna, Austria).

### Halting the trial

The STRIDER Canada trial was halted when the highly-publicised interim analysis of the Dutch STRIDER signaled the potential for harm to the neonate (increased risk in persistent pulmonary hypertension of the newborn) and a non-significant trend towards increased neonatal mortality [11]. Initially, the trial was halted to enable a full and considered review of the contemporaneous evidence, without exposing more pregnancies to potential harm. By then, the UK group had published their results [12] and the STRIDER NZAus group had completed their trial (results shared in confidence) [13]; neither trial had identified any signal of benefit from sildenafil. Consultations were held within the trial steering group, with the UBC Clinical Research Ethics Board, and the DSMB. While no party felt that it was unethical to continue with the trial as the STRIDER Canada was recruiting women earlier, requiring low PlGF as an entry criterion, and not recruiting women with persistent reversed umbilical artery end-diastolic flow, the consensus was that it would be futile to continue given both the publicity and lack of evidence of potential benefit from either the NZAus or UK STRIDER trials. Therefore, the trial was halted.

### Results

Between May 1, 2017 and June 28, 2018, 21 women gave informed consent and were recruited to the STRIDER Canada trial (trial profile, Fig. 1).

At the time of halting the trial, nine women were randomized to the placebo arm and 11 to the sildenafil arm, with one post-randomization withdrawal from the placebo arm prior to commencing placebo medication. No women were actively participating in the trial when it was halted early, as all had delivered.

Recruited women were generally in their early 30 s, nulliparous, overweight, Caucasian, and normotensive; recruitment was at around 22 weeks’ gestation (Table 1). Fetuses were generally very growth restricted with uniform evidence of feto-placental compromise in terms of either abnormal uterine or umbilical artery or ductus venosus Doppler waveforms or critically-low maternal plasma PlGF levels.

**Table 1:** Baseline Characteristics (number (percent); median [interquartile range])

Variable	Placebo (n = 9)	Sildenafil (n = 11)	P-value*
Maternal age at estimated date of delivery (years)	30.0 [29.0, 31.0]	33.5 [31.5, 37.3]	0.109
Nulliparity	5 (55.6%)	6 (54.5%)	0.964
Height (cm)	160.0 [160.0, 165.0]	159.0 [155.5, 165.8]	0.471
Weight (kg)	75.0 [68.7, 77.7]	69.4 [59.0, 83.7]	0.743
Body-mass index	27.1 [24.7, 29.3]	27.6 [22.4, 34.3]	0.543
Ethnicity			0.514
Caucasian	7 (77.8%)	6 (54.5%)	
Afro-Canadian	1 (11.1%)	2 (18.2%)	
South or East Asian	0 (0%)	2 (18.2%)	
Other	1 (11.1%)	1 (9.1%)	
Current smoker	0 (0%)	1 (9.1%)	1.000
Current antihypertensive treatment	0 (0%; chronic hypertension)	1 (9.1%)	1.000
Gestational diabetes	0 (0%; 1 [11.1%] woman subsequently diagnosed)	0 (0%)	0.918
Preterm prelabour rupture of membrane	0 (0%)	0 (0%)	NA
Systolic blood pressure with the highest prerandomization sBP (mmHg)	124.0 [108.0, 132.0]	124.0 [106.5, 136.0]	0.760
Diastolic blood pressure with the highest prerandomization dBP (mmHg)	72.0 [67.0, 75.0]	76.0 [70.5, 84.0]	0.127
Creatinine (μmol/L)	55.5 [53.75, 58.25]	56.0 [49.0, 57.8]	0.831
Uric acid (μmol/L)	265 [253, 278]	220 [210, 250]	0.571
Abnormal uterine artery Doppler ^†^	6 (66.7%)	6 (54.5%)	1.00
Fetal abdominal circumference < 1st percentile for GA	6 (66.7%)	8 (72.7%)	0.659
Estimated fetal weight (g)	301.0 [240.0, 488.0]	246.0 [234.5, 389.5]	0.403
Estimated fetal weight < 500 g	6 (66.7%)	9 (82.0%)	0.432
Umbilical artery Doppler end-diastolic flow			0.537
Intermittently absent	2 (22.2%)	1 (9.1%)	
Persistently absent	3 (33.3%)	2 (18.2%)	
Intermittently reversed	0 (0%)	1 (9.1%)	
Reversed ductus venosus a-wave	0 (0%)	2 (18.2%)	0.600
Maternal PlGF (immediately prior to randomization)	12.0 [12.0, 12.0]	12.0 [12.0, 19.8]	0.447
GA at PlGF collection, eligibility & randomisation (weeks + days)	22^+4^ [21^+5^, 25^+5^]	21^+5^ [21^+4^, 23^+5^]	0.379

^*^ Fisher’s exact (dichotomous) or Mann Whitney U (continuous) test

^†^ Elevated uterine artery pulsatility index for gestational age or persistent unilateral or bilateral notching

There were no differences between arms in the primary outcome (gestational age birth) or markers of maternal or perinatal compromise (Table 2). Of note, the two cases of PPHN occurred in neonates exposed to sildenafil, consistent with the Dutch experience [11].

**Table 2:** Primary, maternal, and perinatal outcomes (number (percent); median [interquartile range])

Outcome	Placebo (n = 9)	Sildenafil (n = 11)	P-value*
Primary outcome			
Gestational age at delivery (weeks + days)	29^+2^ [28^+1^, 34^+1^]	26^+4^ [25^+6^, 31^+0^]	0.200
Maternal			
Symptomatic hypotension	0 (0%)	0 (0%)	NA
Pre-eclampsia	3 (33.3%)	0 (0%)	0.211
Mode of delivery			0.348
Termination of pregnancy	1	4	
Vaginal birth	0	1	
Classical Caesarean section	4	4	
Lower segment Caesarean section	4	2	
Haemorrhage requiring transfusion	1 (11.1%)	0 (0%)	0.918
Perinatal			
Fetal growth velocity			
Biparietal diameter (mm/days)	0.36 [0.32, 0.49]	0.37 [0.26, 0.39]	0.508
Head circumference (mm/days)	1.28 [1.03, 2.07]	1.21 [1.04, 1.52]	0.605
Abdominal circumference (mm/days)	1.05 [0.97, 1.21]	1.37 [1.10, 1.38]	0.310
Femur length (mm/days)	0.28 [0.20, 0.31]	0.24 [0.16, 0.30]	0.627
Estimated fetal weight (g/days)	10.80 [6.07, 11.82]	7.19 [5.62, 9.40]	0.667
Deepest vertical amniotic fluid pocket (mm/days)	0.007 [0.000, 0.009]	0.013 [0.007, 0.070]	0.181
Stillbirth	1 (11.1%)	4 (36%)	0.436
Neonatal death	1 (11.1%)	1 (9%)	1
Intact survival^†^	7 (77.8%)	6 (55%)	0.528
Persistent pulmonary hypertension of the newborn	0 (0%)	2 (18%)	0.164

^*^ Fisher’s exact (dichotomous) or Mann Whitney U (continuous) test

^†^ Defined as survival to estimated due date without evidence of severe central nervous system injury (by ultrasound and/or magnetic resonance imaging)

## Limitations

Halted early in response to safety concerns and no evidence of efficacy, STRIDER Canada was underpowered to provide any meaningful data as an isolated trial. However, because of the a priori planned IPD meta-analysis [10], for which the women signed explicit consent, the data from the trial will inform analysis of the international experience of sildenafil for early-onset FGR.

Although ethical review when the Dutch data were publicised determined that it would be ethical to continue with the STRIDER Canada trial, given that the Canadian trial recruited earlier in gestation than the other trials and was the sole trial that required PlGF < 5th percentile for GA as an eligibility criterion, the level of negative publicity, loss of momentum, and the low recruitment rate along with no signal of benefit in the completed trials to date (NZAus and UK) rendered the trial unfeasible (note: our stopping rule had been two trials reporting adverse outcomes).

The major strength of this undertaking was engagement with varied international funders (Health Research Council of New Zealand, UK National Institute of Health Research, ZonMw [Netherlands], Health Research Board [Ireland (trial halted prior to initiation)], and CIHR) to fund a group of individually-powered trials with primary outcomes that are surrogates of risk, in the knowledge that there was an a priori plan for the ongoing IPD meta-analysis. All funders agreed to the data being centrally managed (UBC PRE-EMPT) using a shared REDCap database on a shared server [10]. We believe that this is a model that should be considered for future obstetric trials to enable adequate sample sizes to examine less common, but more important outcomes. In this instance, the IPD meta-analysis primary outcome is survival to term without evidence of serious adverse neonatal outcome {severe central nervous system injury [severe intraventricular haemorrhage (grades 3 and 4) or cystic periventricular leukomalacia, demonstrated by ultrasound and/or magnetic resonance imaging], or other severe morbidity (bronchopulmonary dysplasia, retinopathy of prematurity requiring treatment, or necrotising enterocolitis requiring surgery)}.

Despite leading the examination of a potential role for sildenafil for severe, early-onset FGR [5], in that original paper we stated that we counselled against sildenafil being prescribed outside either a randomised controlled trial or the perinatal pharmacological surveillance then provided by the Hospital for Sick Children’s Motherisk Program. We are aware that a number of women were prescribed sildenafil for this indication internationally. This should no longer be the case [14].

International consortia can develop, co-fund, and deliver RCTs designed a priori for IPD meta-analyses for important, but uncommon, pregnancy complications. Having been halted early, STRIDER Canada is underpowered to provide any independent guidance about the advisability of sildenafil use in pregnancy. However, the data of those who participated in the STRIDER Canada trial in good faith will contribute meaningfully through the STRIDER IPD.

## Data Availability

Data are being shared with the STRIDER IPD consortium and are available through direct contact with the STRIDER Canada Trial team at STRIDERCanada@cw.bc.ca.

## Abbreviations

AC: Abdominal circumference
BC: British Columbia
CIHR: Canadian Institutes of Health Research
DSMB: Data safety and monitoring board
EMMA: Evaluating maternal markers of adverse placental outcomes
FGR: Fetal growth restriction
GA: Gestational age
GONet: Global Obstetric Network
IPD: Individual participant data
iSTAR: Integrated System for Trial Allocation and Randomisation
NZAus: NZ-Australia
PlGF: Placental growth factor
PPHN: Persistent pulmonary hypertension of the newborn
PRE-EMPT: PREgnancy—Evidence, Monitoring, Partnerships & Treatment
STRIDER: Sildenafil TheRapy in dismal prognosis early onset fetal growth restriction
UBC: University of British Columbia
